# Minimum Intervention in Managing Two Cases of Tetracycline Staining of Different Severity

**DOI:** 10.7759/cureus.21289

**Published:** 2022-01-16

**Authors:** Matheel Z AL-Rawas, Beh Yew Hin, Yanti Johari, Zuryati Ab-Ghani, Adam Husein

**Affiliations:** 1 Prosthodontics Unit, School of Dental Sciences, Health Campus, Universiti Sains Malaysia, Kelantan, MYS; 2 Prosthodontics Unit, Hospital Universiti Sains Malaysia, Kelantan, MYS; 3 Department of Restorative Dentistry, Faculty of Dentistry, Universiti Kebangsaan Malaysia, Kuala Lumpur, MYS

**Keywords:** clinical report, teeth discoloration, extended bleaching, conservative treatment, case report

## Abstract

Patient dissatisfaction with tooth discoloration as a result of tetracycline therapy is not uncommon. To address patients' aesthetic demands, conservative bleaching treatments were considered before more invasive, irreversible treatments such as dental veneers or crowns. Bleaching is a relatively non-invasive, safe, and cost-effective method of achieving a desirable result. However, due to many limiting variables, including the extended duration of active bleaching, tetracycline-stained teeth are one of the most challenging cases to obtain satisfactory bleaching outcomes. This clinical report presents two cases of management of tetracycline staining of the teeth with varying degrees of severity.

## Introduction

Shwachman and Schuster were the first to observe unpleasant tooth discoloration in children with cystic fibrosis owing to tetracycline administration during tooth development [1]. There are still patients who suffer from staining of permanent teeth due to tetracycline treatment when they were children. Tetracycline deposition inside the bone and dental hard tissues is linked to systemic administration of tetracyclines throughout human development since the active component and its homologues have the potential to form complexes with calcium ions on the surface of hydroxyapatite crystals. Tetracycline can pass the placental barrier, causing harm to the developing baby; hence, it should be avoided from 29 weeks until full term to avoid integration into the oral tissues. Furthermore, because permanent teeth continue to form in infants and young children until the age of 12, tetracycline should be avoided in children under the age of 12 as well as breastfeeding and pregnant mothers [2].

In Malaysia, Yaacob et al. found that tetracycline discolored the teeth of 88 (1.96%) of the 4,500 patients. The bulk of the stains was yellowish-brown (59.1%), with 37.5% being greyish-brown and 3.4% being black. Up to two-thirds (79.6%) of the crowns of a substantial number of teeth were discolored [3]. Hegde et al. investigated the prevalence of tetracycline discoloration in anterior teeth in the south Canara population in the years 2012 and 2014. They discovered that the number of people with tetracycline-induced stains increased by 3.8% in 2014 compared to 2012 despite the awareness they created to avoid such a complication [4].

Tetracycline-stained teeth ranged from deep yellow to greyish dark discoloration with or without banding as classified by Jordan and Boksman. It is important to understand how discoloration on the teeth is classified before attempting teeth bleaching, as each different classification posed an increasing complexity of treatment, which entailed minimally invasive treatment to a more invasive full-coverage crown [5]. Mild tetracycline staining was categorized as a first-degree stain. This stain is yellowish to greyish in color, has no banding evident, and is evenly distributed across the tooth. Moderate tetracycline staining was categorized as a second-degree stain. The staining ranges from golden brownish to dark greyish. Severe tetracycline staining was categorized as a third-degree stain whereby it is blue greyish or blackish in color, and there was a considerable banding across the teeth. Intractable staining was the fourth-degree stain, and it is so intense that bleaching was reported to be unsuccessful. The first three classes, mild, moderate, and severe, may often be bleached successfully [6].

Tetracycline caused not only tooth discoloration but also enamel hypoplasia [7]. However, the association between tetracyclines and enamel hypoplasia was unconfirmed as this may be due to other illnesses or interruptions during teeth mineralization [8].

Bleaching is non-invasive, safe, financially sustainable, and able to produce a favorable outcome. However, tetracycline-stained teeth are one of the most difficult cases to achieve acceptable bleaching results due to various limiting factors, especially the long period of active bleaching. This information should be communicated with the patient before whitening tetracycline-discolored teeth [7].

This clinical report presents two cases of tetracycline-stained teeth with varying degrees of severity and presentation. The aim was to discuss the predictability of a non-invasive treatment in managing patients with tetracycline stains. The objective of this paper was to highlight the reliability of home bleaching protocols in managing tetracycline-stained dentition and the potential patient-related limiting factors that may affect the overall outcome of the treatment.

## Case presentation

Case 1

A 34-year-old gentleman requested treatment for his discolored yellowish-brown posterior teeth. The patient and his family could not recall the incidence of tetracycline ingestion; however, they stated the occurrence of multiple episodes of upper respiratory tract infections during childhood. This information together with the characteristic distribution and color of the affected teeth had influenced the diagnosis and the management. The patient was diagnosed with type II diabetes mellitus, which was controlled by an oral hypoglycemic agent. The patient requested to have his discolored teeth be bleached without any invasive procedures.

**Figure 1:**
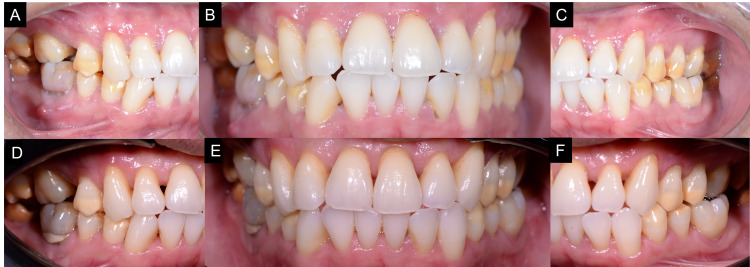
(A) Pre-operative right buccal view. (B) Pre-operative frontal view. (C) Pre-operative left buccal view. (D) Post-operative right buccal view. (E) Post-operative frontal view after seven months of at-home bleaching showing the discolored bands had post-bleaching shades ranging from B3 to A3.5. (F) Post-operative left buccal view.

**Figure 2:**
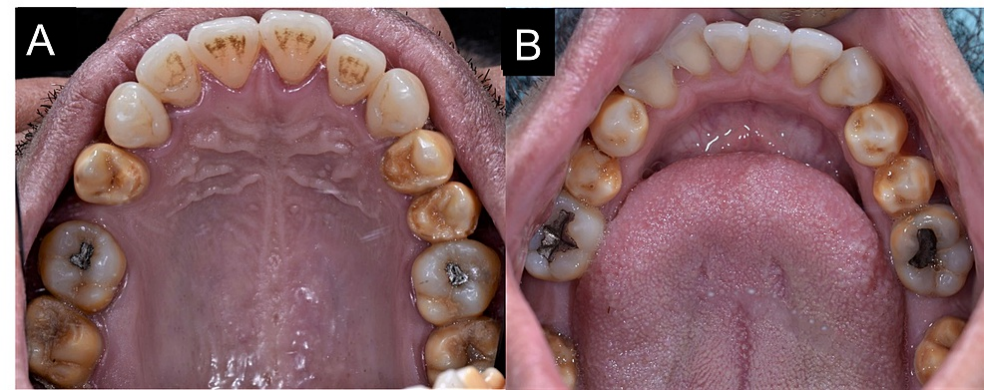
Intraoral maxillary occlusal view of discolored premolar teeth with signs of hypoplasia. (B) Intraoral mandibular occlusal view of discolored premolar teeth with signs of hypoplasia.

Intraoral examinations revealed that all premolars and second and third molars were affected. The anterior teeth were spared. The discoloration showed a yellowish-brown banding over the occlusal and middle thirds of the affected teeth (Figures 1A-1C). The discoloration was more generalized over the mandibular third molars. There were signs of hypoplasia related to the affected teeth clearly visible on the occlusal surfaces of the premolars (Figures 2A, 2B); however, this was not confirmed as this may be due to other illnesses or interruption during mineralization for which the tetracycline had been originally prescribed [8]. No invasive treatment was applied as the patient was not concerned about the hypoplasia, and a patient-centered approach was followed in this case.

**Figure 3:**
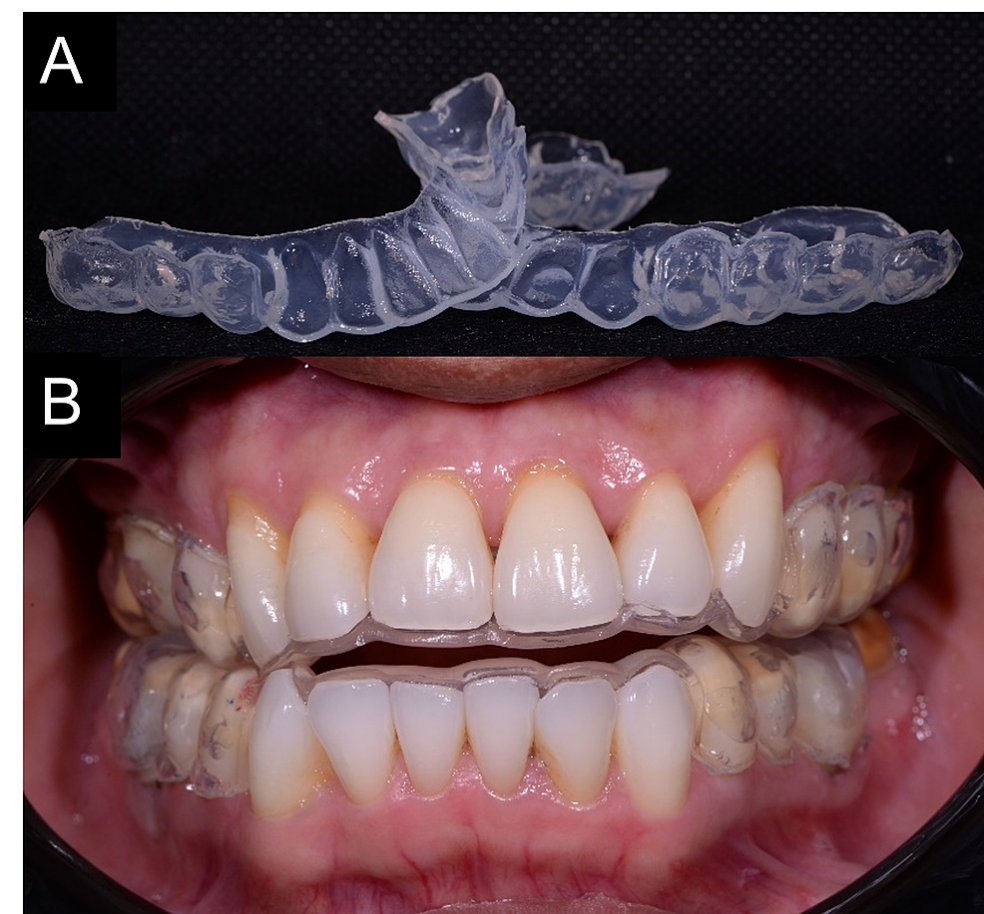
(A) Prepared bleaching trays. (B) Bleaching trays were fitted inside the patient’s mouth.

After a detailed discussion of different treatment options and their advantages and disadvantages, the patient agreed to have at-home bleaching done on all maxillary and mandibular premolars alone by using a customized bleaching tray. After the preventive treatment and endodontic treatment of tooth 46, impressions were made with alginate (Kromopan®, Lascod, Italy), which were then sent to the laboratory to construct a thermoplastic bleaching tray (Erkoflex®, Erkodent, Germany) of 1 mm thickness and cut to scalloped margins with reservoirs. Afterward, the trays were fitted over the maxillary and mandibular arches and checked for fit and for any possible trauma to the gingiva (Figures 3A, 3B). A 10% carbamide peroxide (CP) (Opalescence PF 10%, Ultradent Inc., South Jordan, UT, USA) kit was given to the patient as the discoloration degree was of moderate severity. The patient was advised to wear them for at least six hours overnight for three months initially and to be reviewed monthly until satisfactory result.y. The patient was advised to wear it for at least six hours overnight for three months initially and to be reviewed monthly until satisfactory result. The Vita-pan classical shade guide and spectrophotometer (Vita Easyshade®, Vita-Zhanfabrik, Bad Säckingen, Germany) were used to assess the teeth shades.

The discolored bands' pre-bleaching shades were C4, as shown in Figure 1B, and after seven months of treatment, their shades were between B3 to A3.5 (Figures 1 D-1F). The tetracycline-stained regions were found to be bleached with some improvement compared to the pre-bleaching state. The patient had minor sensitivity that did not result in impairment with his regular activities. This sensitivity was managed by using desensitizing toothpaste (Sensodyne repair and protect, GSK, Selangor, Malaysia). However, there was an issue with the compliance because the patient stated that he could not wear the bleaching tray on a regular basis. Because of his time restrictions, the patient was happy with the results and declined to continue with the bleaching procedure. Due to COVID-19 restrictions, the patient did not attend the review appointments.

Case 2

A 32-year-old dental postgraduate resident was referred to our prosthodontic postgraduate clinic with a concern of teeth discoloration. He was aware that his teeth were stained by tetracycline. Both medical and dental history was non-contributory. He had no known history of allergic reactions toward any particular drugs, food, or chemical substance. He did not know the timing and dose of his tetracycline exposure, and his parents failed to recall such incidence. He strictly refused any restorative treatment to manage his condition and only considered non-invasive treatment options.

**Figure 4:**
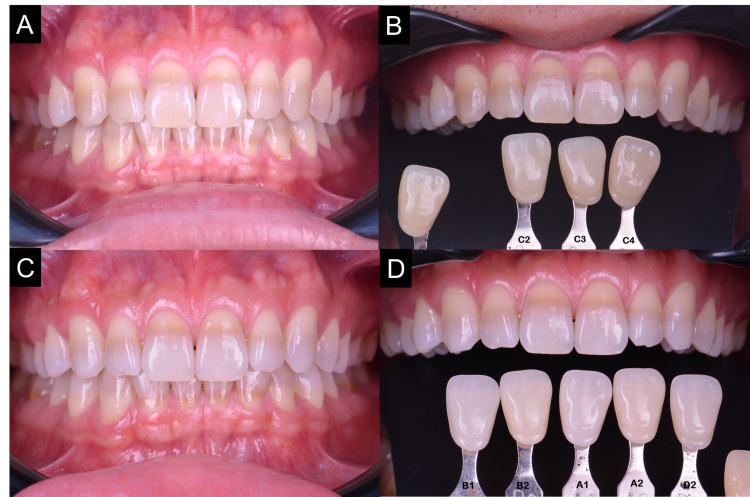
(A) Pre-operative frontal view. (B) Pre-operative shade of C2. (C) Frontal view after six months of at-home bleaching. (D) Post-operative shade of A1 was achieved and retained; however, the band discoloration still persist.

Intraoral examinations revealed a yellowish-brown discoloration with a dark brown band affecting all his maxillary and mandibular anterior dentition and all four first permanent molars sparing the premolars (Figures 4A, 4B). The discoloration appeared at the cervical third of central incisors, moving more coronally as it approached the lateral incisors and canines, while on the first molar, it was isolated on the cervical third. Otherwise, the patient had an unrestored dentition with the basic periodontal examination (BPE) scored 2 at the mandibular anterior sextant.

The patient agreed to have at-home bleaching done on all the maxillary and mandibular anterior and premolars with a customized tray to manage the discoloration. The first molars were minimally affected by the discoloration; hence, bleaching was not required. Furthermore, it was not significantly visible upon smile analysis. After the non-surgical periodontal therapy, an impression was made using alginate (Kromopan), which was poured with dental stone. A custom bleaching tray was constructed with a 1.0-mm heat-pressed soft thermoplastic sheet (Erkoflex) with a scalloped, reservoired tray design. Using 16% CP (Opalescence PF 16%, Ultradent Inc.), the patient was advised to wear it for at least four to six hours overnight for three months initially and to be reviewed monthly until satisfactory result. In any incidence of dentinal hypersensitivity, the patient was advised to temporarily halt the bleaching procedure and was provided with GC Tooth-Mousse Plus (GC Corporation, Tokyo, Japan) to be applied using the bleaching tray. In this case, 16% CP bleaching was chosen considering the severity and location of the discoloration. This was the highest concentration of CP available in our country due to the local regulations.

The pre-bleaching shade was at shade C2 (Vita classical, Vita-Zhanfabrik) (Figure 4B) and progressed to shade A1 (Figures 4C, 4D). It was noticed that the tetracycline-stained area was not effectively bleached, but the overall teeth shade improved significantly. There was no reported issue with dentinal hypersensitivity. The patient expressed his satisfaction toward the final shade attained and did not wish to proceed further. The subsequent four months post-bleaching review showed shade retention at A1.

## Discussion

Response to bleaching varies among patients and the rate of bleaching differs individually, and this makes it worse for the tetracycline-stained teeth [7,9]. Even so, the current literature cited that a reasonable outcome was achievable [7,10,11]. Despite the suboptimal bleaching outcome on the deeply stained dark brown band discoloration explicit in case 2, the overall shade had improved tremendously. The patient expressed a high level of satisfaction and comfort throughout the treatment duration. In case 1, the yellowish-brown bands were successfully bleached to a certain limit, which pleased the patient. The bleaching results could have been better; however, due to the patient's lack of compliance, the outcomes were deemed limited.

Location of discoloration in the arch and in the tooth itself depends on the patient’s age and teeth calcification at the time and duration of tetracycline administration. Deposition of the tetracycline may be continuous or laid down in stripes depending on whether the ingestion was continuous or interrupted [2]. Teeth calcification time is the reason why the premolars were affected, and anterior teeth were spared in case 1 as well as the reason why only anterior teeth were affected in case two, while posterior teeth were spared except for the first molars. Incisors and canines have an earlier calcification time (calcification starts around 3-12 months of age) compared to premolars (calcification starts around two years of age). The second and third molars have a later calcification time compared to the other teeth.

CP is a chemical compound of urea and hydrogen peroxide (HP) that dissociates back into HP and urea when dissolved in water or saliva. As a result, CP can be considered a precursor of HP, the active bleaching agent. Their activity improves the shade of the stained tooth structure by converting peroxide into free radicals, which break down large, pigmented molecules. The chromophores in these large molecules are responsible for the color stain in the enamel because they absorb light in the visible area. Free radicals break down these molecules into smaller molecules, affecting light absorption and therefore decreasing or eliminating the stain [12]. Because of the chemical compound's nature, it is suggested that 10% or 16% CP be used overnight for optimum effectiveness. CP can stay active for up to 10 hours, with the first two hours responsible for over half of the peroxide released. If overnight wear is not possible or preferred by the patient, daytime wear of two to four hours can be selected [13].

A nightguard vital bleaching study reported a 95% efficacy rate for non-tetracycline discolored teeth and 75% efficacy for tetracycline-stained teeth following a six-week regimen using a 10% CP solution. The same study also showed that patients with tetracycline-stained teeth did not experience the same degree of bleaching as those with other stains, nor did the bleached color of the tetracycline-stained teeth match the degree of whitening achieved in the teeth stained by other causes [14]. Another study on at-home bleaching with extended treatment time (six months) achieved an acceptable result of six patients out of 10 patients. The tetracycline-stained teeth were of moderate-to-severe category. It was noted in the same study that if the neck of the tooth (gingival third) was severely discolored, the bleaching prognosis was reduced [15].

In the abovementioned cases, case 1 utilized 10% CP, while in case 2, 16% CP was prescribed, both were overnight tray bleaching. The different percentage of CP in this report was used to allow an assessment of the possibility to enhance treatment duration. It was also noted that if the neck of the tooth (gingival third) was severely discolored, the prognosis for lightening the complete tooth was reduced. Conversely, if the neck of the tooth was only mildly discolored, a more clinically acceptable result was achieved [15].

The prognostic indicator in tetracycline-stained teeth was not the severity of discoloration but instead the location of the discoloration. As in case 2, the location of the staining together and the darkness of the staining influenced the decision of utilizing a higher concentration home bleaching agent, although one study showed no differences obtained when using 10 or 15% CP in the outcome of the treatment [16].

Case 1 reported mild sensitivity, which did not result in incapacitation, and was able to perform the daily tasks normally, while case 2 did not report any issue with sensitivity. This sensitivity may be related to rapid transenamel and transdentinal diffusion of HP that has low pH to the pulp or other toxic components released with the degradation of the bleaching gels [17]. The previous study did not report any significant sensitivity regardless of when bleaching was done using low-concentration CP as compared to high-concentration HP [11]. Opalescence PF, in both concentrations, contained potassium nitrate (0.5%) and fluoride ions (0.11%) as protective mechanisms integrated into the system. Both active ingredients reduce the incidence of teeth sensitivity and provide adequate fluoride for remineralization. The common strategies to manage teeth sensitivity during bleaching was to use 5% potassium nitrate, desensitizing toothpaste [7], or casein phosphopeptide-amorphous calcium phosphate with fluoride (CPP-ACPF) [18]. The application of CPP-ACPF during or after bleaching resulted in a lesser reported tooth sensitivity [18]. CPP-ACPF can be applied into the bleaching tray and seated onto the teeth for at least 30 minutes or overnight or simply applied to the teeth with a toothbrush or finger without rinsing thereafter.

Overnight tray bleaching with CP compound was proven to result in a more color change [10] and superior comfort [11]. Due to the long-acting time for the urea-stabilized CP, it was shown to be more efficient [10]. However, it shall be noted that other bleaching material and delivery systems, for example, in-office power bleaching with high-concentration HP, daytime strip bleaching with 6% HP, or even a high concentration of 45% CP overnight tray bleaching, do produce a reasonable outcome [10,11]. The decision regarding the bleaching material and its delivery system should not be solely based on the practitioner but consideration shall be taken on the patient’s opinion, compliance level, lifestyle and motivation, and patient-centered approach in decision-making to ensure an optimal outcome.

According to Dubal and Porter, there was no reported difference in efficacy with or without reservoirs, and no teeth suffered the loss of vitality with only reported tooth sensitivity and gingival irritation after and during bleaching [8]. Also, they reported no efficacy difference between extended and non-extended bleaching trays. For these patients, the bleaching tray was designed with a non-extended tray with a reservoir. Open tray design or window technique was advocated when specific teeth were not in the intention for bleaching. This design was described by Haywood and Sword [9]. Occlusal changes are unexpected while using the open tray design. The wearing time is about six to eight hours at night or during the day time, and the normal occlusal forces would correct any unintentional tooth movement when partial coverage appliances were used [19].

The final results of at-home bleaching were influenced by a number of factors. Aside from the degree and location of tooth discoloration and the bleaching type, chemical concentration of bleaching agent, and the duration of treatment, additional factors such as patient compliance, age, tooth sensitivity, and dental hygiene may influence the final outcomes [20,21].

Patients who were older and had a less yellowish initial tooth color had the least mean color change after bleaching, whereas those who were younger and had a more yellow initial tooth color had the highest mean color change after bleaching [21].

Other treatment options other than bleaching of tetracycline staining, such as composite or ceramic veneers, are available; however, they are more invasive and come with a higher maintenance. Both patients were satisfied with the bleaching modality and refused other treatment options.

## Conclusions

In cases of tetracycline-stained dentition, it is critical, to begin with, non-invasive treatment, particularly in patients with minimally restored dentition to protect such healthy tissues from the vicious cycle of the restorative treatments. This case report demonstrated that two cases of varying discoloration severity and distribution can be treated by bleaching procedure with satisfactory results. Achieving the best outcomes is still a challenge because of the many variables that restrict bleaching effectiveness, such as patient compliance and the lengthy time required for active bleaching. Therefore, restorative treatments may be planned with absolute care when necessary.
